# Changes in roller skiing economy among Nordic combined athletes leading up to the competition season

**DOI:** 10.3389/fspor.2024.1320698

**Published:** 2024-03-04

**Authors:** Takuya Yanaka, Mariko Nakamura, Kaoru Yamanobe, Yusuke Ishige

**Affiliations:** ^1^ Department of Sports Science and Research, Japan Institute of Sports Sciences, Tokyo, Japan; ^2^ Faculty of Commerce, Yokohama College of Commerce, Kanagawa, Japan

**Keywords:** aerobic, anaerobic, cross-country skiing, ski jumping, ⩒O_2max_

## Abstract

The purpose of this study was to compare roller skiing economy during different training phases in Nordic combined (NC) athletes and determine the aerobic and anaerobic factors responsible for changes in skiing economy. Seven elite NC athletes underwent incremental load tests on a large buried treadmill in both spring and autumn using roller skis. Measurements included oxygen uptake, respiratory exchange ratio, and blood lactate concentration. Roller skiing economy was calculated from aerobic and anaerobic energy system contributions, and overall roller skiing economy was determined by combining the two. Comparisons were made between the skiing economies obtained in the two measurement sessions. Physical characteristics and incremental test performance remained consistent between the two measurement sessions. The overall skiing economy at each speed significantly improved toward the competition season (*p* < 0.05). Similarly, the contribution of anaerobic energy system at each speed showed significant improvement (*p* < 0.05). In contrast, the contribution of aerobic energy system did not change between the two measurement sessions. This study reveals that NC athletes enhance their skiing economy at the same speed during submaximal efforts in preparation for the competition season. This improvement is predominantly associated with an improvement in the contribution of anaerobic energy system.

## 1 Introduction

In Nordic combined (NC), an athlete's competition ranking is determined by their performance in ski jumping (SJ) and cross-country skiing (XC). SJ requires high vertical jumping power, light body weight, and precise posture control during take-off (i.e., control of angular momentum) (1). Training for SJ focuses on enhancing lower limb power and the timing of power exertion during high-speed descents. Conversely, XC demands both high aerobic and anaerobic capacities (2) as well as efficient gliding (3). Improving these factors necessitates substantial endurance training. However, NC athletes have limited time available for each discipline compared with athletes specializing in SJ or XC (4), which requires them to train efficiently in both areas.

Maximal oxygen consumption $\left(\dot{V}O_{2\max}\right)$ is commonly used as an indicator of aerobic capacity in long-distance events, including XC. Numerous studies have shown a positive correlation between higher $\dot{V}O_{2\max}$ and performance in XC athletes (5–7). Some studies investigating XC athletes using running have observed seasonal variations in $\dot{V}O_{2}$ max (5, 8, 9). Meanwhile well-trained athletes may improve $\dot{V}O_{2\max}$ with high volume intensive training, improvements in $\dot{V}O_{2\max}$ may reach a plateau when training volumes are low (9). As mentioned earlier, NC athletes have less time for endurance training than XC athletes. These suggest that NC athletes may have limited opportunities to enhance their $\dot{V}O_{2\max}$ compared with XC athletes.

Moreover, XC races entail high intensities, particularly during uphill sections on undulating terrain (2), necessitating a highly developed anaerobic capacity. Sandbakk et al. (7) highlighted the importance of anaerobic energy mechanisms in differentiating competition levels. Therefore, supplying adequate aerobic and anaerobic energy is crucial for XC racing. Additionally, efficient gliding with minimal energy expenditure is vital. Oxygen uptake at a given velocity (O_2_-cost) (6) and gross efficiency (3, 7) have been used as methods to measure gliding efficiency. Studies have found that XC athletes with lower energy expenditure exhibit higher performance levels (3, 6, 7). Furthermore, Losnegard et al. (2) demonstrated an improvement in O_2_-cost leading up to the competition season, indicating that XC athletes ski more efficiently during off snow season preceding the competition.

Another measure of efficiency is exercise economy, which quantifies energy consumption per unit distance. Kyröläinen et al. (10) and Tanji et al. (11) calculated running economy by combining aerobic energy expenditure, calculated from oxygen intake and respiratory quotient, with anaerobic energy, as indicated by blood lactate concentration, which is then converted to aerobic energy expenditure. The calculation also has been used to compare roller skiing economy of XC athletes among skiing techniques (12). This index is considered possible to examine the contribution of aerobic and anaerobic energy systems for skiing economy. Previous studies have primarily focused on the efficiency of XC athletes, raising questions about whether similar findings can be applied to NC athletes. Understanding the characteristics of changes in roller skiing economy among NC athletes for each energy mechanism could potentially lead to efficient XC training in the context of SJ training.

The purpose of this study was to compare roller skiing economy between different phases in NC athletes and identify the aerobic and anaerobic factors responsible for changes in roller skiing economy. XC and NC athletes often use roller skis in their XC training during the off-snow season. The sub-technique examined in this study revealed slight differences in hip angle (8°) and pole push time (0.04 s) between roller skis and on snow. However, due to the similarity observed in ground-contact, hip acceleration, and hip displacement patterns, it is suggested that roller skiing can simulate movements on snow (13). Previous studies (14, 15) have reported performance improvements in XC athletes leading up to the competitive season. Losnegard et al. (14) highlighted the enhancement of O_2_-cost and O_2_-deficit, indicators of skiing economy during submaximal intensity exercise, as factors contributing to this improvement. NC athletes also aim to improve their performance for the competitive season, and endurance training is expected to influence these factors. Therefore, we hypothesize that the roller skiing economy of NC athletes will improve in the next competition season, driven by changes in both aerobic and anaerobic factors.

## 2 Methods

### 2.1 Subjects

Seven elite NC athletes participated in this study. They were part of the Japanese national team and competed in international competitions. The physical characteristics of the subjects were age 24.1 ± 6.9 years-old, height 1.72 ± 0.02 m, weight 63.0 ± 3.2 kg, fat free mass (FFM) 56.3 ± 3.0 kg in spring (June) and age 24.6 ± 6.9 years-old, height 1.72 ± 0.02 m, weight 63.8 ± 3.8 kg, FFM 56.4 ± 2.5 kg in autumn (September–October). FFM was measured using the BOD POD Body Composition System (Life Measurement Instrument, California) employing air-displacement plethysmography. There were no differences in physical characteristics between two measurements. Prior to the measurements, the subjects were informed about the study details and their written consent to participate was obtained. The measurement sessions were conducted with the approval of the ethics committee affiliated with the Japan Institute of Sports Sciences.

### 2.2 Experimental procedure

The subjects performed an incremental load test using roller skis (MS610AS, MARWE, Finland) on a large buried treadmill (S3040, ForceLink, Netherlands). Before the test, the subjects underwent a warm-up period consisting of stretching and gliding on the treadmill at their desired speed for approximately 10 min. The test began at 8 km/h on a treadmill with a fixed incline of 5.2% (approximately 3 degrees), and involved gradually increasing the speed by 1.5 km/h every 3 min. During the initial three stages, when the speed was relatively slow, the subjects were allowed to choose sub-techniques freely. However, from the fourth stage onward, they were instructed to use only gear three (G3) technique. This technique involves a synchronized double-poling movement for each skating stroke and is a symmetrical technique commonly adopted on flat to gently uphill terrains, frequently used even in competitions. Additionally, the subjects were given a 1-min break between stages for blood sampling, and the test continued until exhaustion. The test concluded when two of the following criteria were met: (1) the respiratory exchange ratio (RER) exceeded 1.15, (2) the subject reached their age-predicted maximal heart rate (HR) (i.e., 220 – age), or (3) their blood lactate concentration (La) exceeded 8.0 mmol/L (16).

Respiratory gas analysis was performed continuously on a breath-by-breath basis using the computerized standard open circuit technique and an expiratory gas analyzer (AE310-S, Minato Medical Science, Japan). Before and after the measurements, the analyzer was calibrated using two calibration gases (air equivalent: O_2_, 20.90%, CO_2_, 0.03%, N_2_ balance; exhaled air equivalent: O_2_, 15.90%, CO_2_, 5.00%, N_2_ balance). The hot wire flowmeter was calibrated before the experiment using a flow calibrator. The oxygen uptake $\left(\dot{V}O_{2}\right)$ and RER for each stage were averaged over a 1-min period preceding the stage's end. $\dot{V}O_{2\max}$ was determined as the maximum 30-s average $\dot{V}O_{2}$ throughout the test. Blood samples were drawn from the participants' fingertips before the start of the test, immediately after the end of each stage, and immediately after the test's completion. Blood La was measured using two blood lactate analyzers (Lactate Pro2, Arkray, Japan), and the average of the two values obtained was used as the representative value for each point.

Additionally, the HR during the test was measured at a frequency of 12 Hz using a HR monitor (RS800, Polar, Finland). The time to exhaustion (TTE) was defined as the duration until the test was terminated, the HR immediately prior to exercise cessation was defined as the maximum HR (HR_max_), and the blood La immediately after the test was defined as the post-exercise blood La (La_pe_). V_peak_ was calculated based on the method of a previous study (17).

### 2.3 Data analysis

The analysis focused on the period from stage 4 (12.5 km/h), when the subjects started using the G3 technique, to stage 8 (18.5 km/h), at which point all subjects had completed the test. The roller skiing economy was calculated using methods described in previous studies (10–12). An energy equivalent of 20,202 J・ml^−1^ oxygen was applied when RER was 0.82. The change of ±0.01 in RER caused the respective 50 J changes in energy expenditure (18). For example, if RER was 1.00, the energy equivalent applied was 21,102 J・ml^−1^ oxygen. This was the contribution of the aerobic energy systems (C_AE_). When blood La exceeded 2.0 mM (<2.0 mM was negligible), The contribution of anaerobic energy sytem (C_AN_) was then calculated based on an equivalent of 60 J・kg^−1^・mM^−1^ (3 ml O_2_・kg^−1^・mM^−1^) (19). Each energy equivalents were divided by the speed of each stage to obtain C_AE_ and C_AN_. Finally, The overall roller skiing economy (E_ALL_) was determined by combining C_AE_ and C_AN_, the formulas of which were as follows (Equations 1, 2),

(1) $$C_{\mathrm{AE}}\;=\;\frac{20202-(0.82-\mathrm{RE}R_{\mathrm{stage}})\;\times \;50\;\times \;100\;\times \;\dot{V}O_{2\;\mathrm{stage}}}{V_{\mathrm{stage}}},$$

(2) $$C_{\mathrm{AN}}\;=\;\frac{60\;\times \;(La_{\mathrm{stage}}-La_{\mathrm{rest}})}{V_{\mathrm{stage}}\times 3},$$

where _stage_ represents the value of each stage, _rest_ represents the value before the start of the test, and V_stage_ represents the speed of each stage.

### 2.4 Statistical analysis

Means and standard deviations were calculated for each parameter in the incremental load test. The normality of each parameter was tested using the Shapiro-Wilk test. With the exception of stage 7 of spring C_AE_, the other parameters were shown to be normally distributed (*p* > 0.05). Paired t-tests were conducted to determine differences between seasons for the parameters in the incremental load test, and t-values and Cohen's d were calculated as effect size (ES). Furthermore, to compare the roller skiing economy (E_ALL_) and the contributions of aerobic and anaerobic energy systems (C_AE_, and C_AN_) at each stage between seasons, a correlated two-factor analysis of variance (ANOVA; 5 stages × 2 seasons) was employed to test for interactions and main effects. In cases where interaction was found, a *post hoc* test using the Bonferroni method was conducted to assess simple main effects. For the ANOVA, *F*-values and partial *η* were calculated as ES. The significance level for all tests was set at 5%. All data analyses were conducted using statistical software (SPSS 24.0 for Windows, IBM, Japan).

## 3 Results

No significant differences were observed in incremental load test performance (Table 1).

**Table 1:** Performance parameters of the incremental load test.

		Spring	Autumn	*p*	*t*	ES
$\dot{V}O_{2\max}$	(ml/kg/min)	64.4 ± 3.2	64.8 ± 2.0	0.74	−0.35	0.13
La_pe_	(mM)	17.6 ± 3.0	15.6 ± 1.9	0.19	1.49	0.77
HR_max_	(b/m)	192.3 ± 6.8	190.0 ± 6.8	0.21	1.40	0.34
TTE	(min)	33.7 ± 1.8	35.3 ± 2.7	0.10	−1.97	0.72
V_peak_	(km/h)	19.3 ± 0.6	19.9 ± 0.9	0.07	−2.17	0.75

**Figure 1:**
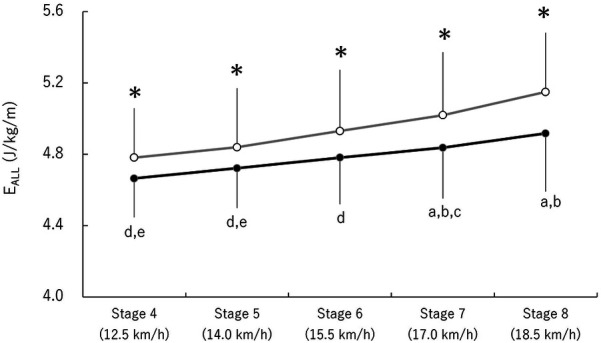
Changes in E_ALL_. Open and closed circles represent spring and autumn data, respectively. Lowercase letters indicate significant differences between stages: (a) vs. Stage 4, (b) vs. Stage 5, (c) vs. Stage 6, (d) vs. Stage 7, and (e) vs. Stage 8. *Represents a significant difference between seasons (*p* < 0.05).

A significant main effect of the season [*F* (1,6) = 6.97, *p* = 0.04, ES = 0.54] and stage [*F* (4,24) = 20.34, *p* < 0.001, ES = 0.77] was found for E_ALL_ (Figure 1), with no significant interaction between the two factors [*F* (4,24) = 0.74, *p* = 0.58, ES = 0.11]. E_ALL_ in autumn was consistently lower than in spring across all stages. Additionally, E_ALL_ significantly increased in stage 7 and 8 compared with stage 4 and 5 (*p* < 0.05).

**Figure 2:**
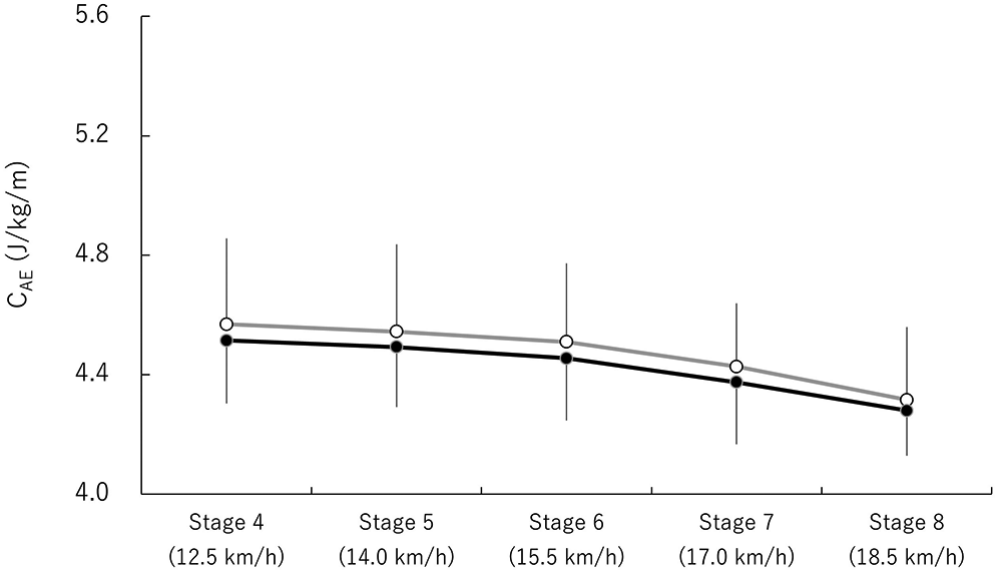
Changes in C_AE_. Open and closed circles represent spring and autumn data, respectively.

A significant main effect of stage [*F* (1.6,9.6) = 10.53, *p* < 0.001, ES = 0.64] was observed for C_AE_, with no significant interaction between the two factors [*F* (4,24) = 0.04, *p* = 0.99, ES = 0.01]. However, no significant main effect of season was found for C_AE_ (Figure 2).

**Figure 3:**
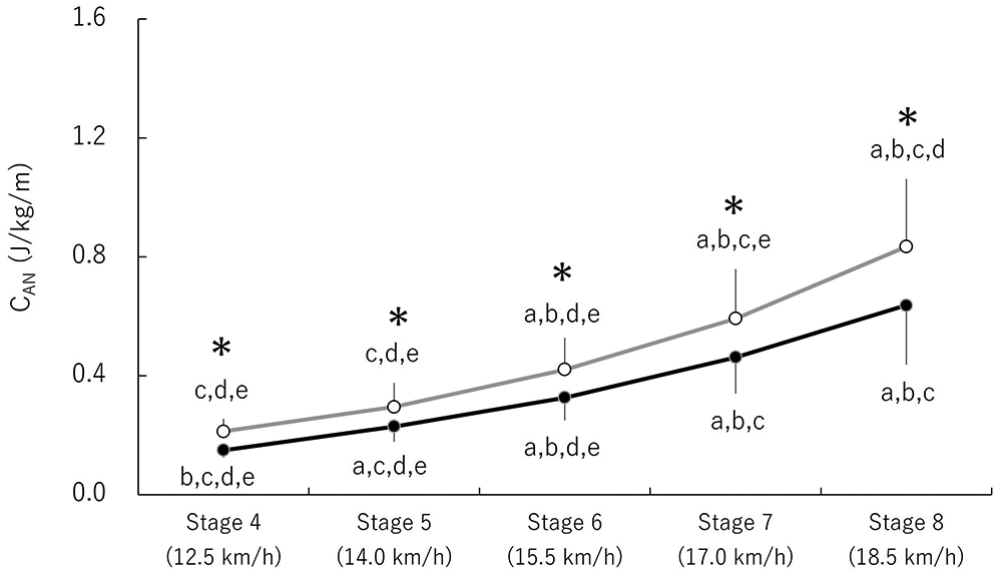
Changes in C_AN_. Open and closed circles represent spring and autumn data, respectively. Lowercase letters indicate significant differences between stages: (a) vs. Stage 4, (b) vs. Stage 5, (c) vs. Stage 6, (d) vs. Stage 7, and (e) vs. Stage 8. *Represents a significant difference between seasons (*p* < 0.05).

Significant main effects of season [*F* (1,6) = 26.91, *p* < 0.001, ES = 0.82] and stage [*F* (1.5,8.8) = 55.81, *p* < 0.001, ES = 0.90] were observed for C_AN_, along with a significant interaction between the two factors [*F* (2.1,12.8) = 4.29, *p* = 0.04, ES = 0.42] (Figure 3). C_AN_ in autumn was consistently lower than in spring across all stages. In spring, C_AN_ did not differ between stage 4 and 5 but significantly increased in later stages (*p* < 0.05). In contrast, in autumn, C_AN_ significantly increased from stage 4 to stage 7 but showed no difference between stage 7 and 8 (*p* < 0.05).

## 4 Discussion

The aim of this study was to examine changes in roller skiing economy among high-level NC athletes during different seasons leading up to the competition. The results revealed that E_ALL_ decreased as the competition season approached, and this was attributed to an improvement in anaerobic energy system. These findings support the hypothesis that roller skiing economy improves as competition approaches, but we must partially reject the hypothesis that both aerobic and anaerobic factors contribute to the improvement.

No significant changes in physical characteristics were observed between each measurement. NC athletes must also perform SJ, and extreme changes in body composition, such as increases in body weight and fat-free mass (FFM), can negatively impact SJ performance. Additionally, there were no statistically significant changes in roller skiing performance (Table 1), but moderate ES was observed for La_pe_, TTE, and V_peak_. Some studies have reported improvements in performance measures, including TTE, over the competitive season, whereas aerobic capacity measures, such as $\dot{V}O_{2\max}$, remained unchanged (14, 15). Although statistically significant changes were not observed in the present study, the trends were similar to those reported in previous studies. The small sample size may have contributed to the lack of significant changes.

E_ALL_ decreased at submaximal speeds leading up to the competition season (Figure 1), suggesting that NC athletes were able to glide more efficiently. This is consistent with findings from a previous study, in which XC athletes reported improved efficiency after three months of daily training (14). Considered in the context of decreasing E_ALL_, significant changes were observed in C_AN_ at submaximal speeds, whereas C_AE_ did not change at such speeds between each test. These results indicate that the change in E_ALL_ was driven by changes in C_AN_ rather than C_AE_. A previous study (8, 9) suggested improved anaerobic energy system as a factor contributing to enhanced endurance performance for well-trained athletes during training periods rather than aerobic energy system, which is consistent with the present findings. It is possible that the adaptation of anaerobic energy system occurs more rapidly than that of aerobic energy system.

C_AN_ was calculated from blood La. Blood La is also the value resulting from its production by the glycolytic (anaerobic) system and its removal by oxidative (aerobic) system (20). When considering factors affecting improved skiing economy, these include changes in kinematics (e.g., cycle time and length) (21), muscle fiber–type alterations, increased buffering capacity within the muscles (22), enhanced oxidative capacity through mitochondria (23), and increased capillary density (24). Considering that C_AE_ and $\dot{V}O_{2\max}$ did not improve, it is likely that the ability of mitochondria and capillaries to remove blood La through relevant oxidative system was not altered. It is also difficult to assume that NC athletes undergo changes in muscle fiber-type, given that they perform endurance training for XC and power training for SJ. Importantly, as the subjects in this study were well-trained, adaptations in physiological areas may occur at a slower pace. Therefore, changes in blood La concentrations are not likely to be due to oxidative system, but to depend on the generation of the glycolytic system. The changes in kinematics are considered the most likely reason for the reduced blood La production and improved contribution of anaerobic energy system. Moreover, kinematics can be modified through short-term training, suggesting that kinematics may be the primary reason for the reduced roller skiing economy associated with C_AN_. Indeed, it has been reported that changing kinematics can alter the blood La (25, 26). Longer and more extensive training may be necessary to improve factors other than kinematics in the well-trained NC athletes in this study (14). However, NC athletes have limited time for endurance training owing to their involvement in SJ. For instance, compared to the training time and sessions of XC athletes, NC athletes only devote about 70% of that of XC athletes to cross-country ski training (72% of training hours and 77% of sessions) (4). Hence, the lack of improvement in aerobic system was an expected outcome.

### 4.1 Limitation

A limitation of this study was the small sample size (*n* = 7), which may have influenced the statistical results. However, it should be noted that the subjects were highly competitive athletes participating in international-level competitions, making these data valuable for the development and enhancement of NC athletes. As noted above, the blood lactate concentration used to calculate C_AN_ is measured as the net of production and removal during exercise (20). The exercise of each stage in this study was submaximal intensity, not maximal intensity. The response of the oxidative system reached a steady state after 2–3 min, suggesting that the removal of blood La was constant. The production of blood La by the glycolytic system did not change significantly because it was also submaximal intensity. Therefore, blood lactate levels were considered stable, given that both systems were at steady state, although blood La fluctuated fluidly between production and removal. In addition, although this study focused solely on roller skiing economy, changes in economy are likely associated with kinematics and training. These aspects should be addressed in future research.

### 4.2 Practical application

This study revealed that physical characteristics and physiological indices at maximum intensity, such as $\dot{V}O_{2\max}$, La_pe_, and HR_max_, as well as roller skiing economy associated with the contribution of aerobic energy system, remained unchanged over a three-month training period. However, the contribution of the anaerobic energy system changed during the training period. To enhance the skiing economy of NC athletes, two approaches are suggested: (1) making short-term technical adjustments, such as cycle length and cycle time, to improve the anaerobic energy system, and (2) implementing long-term training to enhance factors related to metabolism and improve the aerobic energy system. It is important to tailor the strategies for improving economy based on the athletes' level, with short-term improvements focused on elite athletes and long-term improvements aimed at developing athletes in lower categories.

## 5 Conclusion

In NC athletes, overall roller skiing economy decreased consistently at submaximal speeds leading up to the competition season. This decline was primarily attributed to an improvement in anaerobic energy system.

## Data Availability

The raw data supporting the conclusions of this article will be made available by the authors, without undue reservation.
